# Patient safety knowledge, attitude and practice among undergraduate health science students in South West Ethiopia

**DOI:** 10.3389/fpubh.2022.1064896

**Published:** 2023-01-09

**Authors:** Tofik Mohammed, Emebet Woldearegay, Shemsu Kedir, Kemal Ahmed, Masrie Getnet, Esayas Kebede Gudina

**Affiliations:** ^1^Department of Internal Medicine, College of Medicine and Health Science, Arbaminch University, Arba Minch, Ethiopia; ^2^Department of Internal Medicine, Institute of Health, Jimma University, Jimma, Ethiopia; ^3^Department of Public Health, College of Medicine and Health Science, Werabe University, Werabe, Ethiopia; ^4^Department of Public Health, School of Public Health, College of Medicine and Health Science, Wollo University, Dessie, Ethiopia; ^5^Department of Public Health, Institute of Health, Jimma University, Jimma, Ethiopia

**Keywords:** patient safety, knowledge, attitude, practice, undergraduate students, Ethiopia

## Abstract

**Background:**

Patient safety is minimizing the risk of unnecessary damage associated with healthcare to a minimum. It has been linked as a global precedence area where substantial knowledge gaps exist. Knowledge, attitude, and practice of the healthcare providers toward patient safety have a great influence on the delivery of safe patient care. Regardless of this, the issue has not been adequately addressed in Ethiopia.

**Objective:**

The main aim of this study was to assess the knowledge, attitude, practice, and factors associated with patient safety practice among undergraduate health science students at Jimma University Institute of Health.

**Methods:**

An institution-based cross-sectional study design was conducted from May to November 2021. Data were collected from 678 undergraduate health science students using a pretested, structured and self-administered questionnaire. EPI data 3.1 was used for data entry, and SPSS version 25 was used for analysis. A binary logistic regression model was utilized to identify factors associated with outcome variables. An adjusted odds ratio with a 95% confidence interval and *P* < 0.05 were computed to determine the level of significance.

**Results:**

Of the total students, only 293 (43.2%) and 308 (45.4%) had good knowledge and positive attitudes toward patient safety, respectively. Moreover, only 135 (19.9%) of the students had good practices regarding patient safety. Year of study [AOR = 3.75, 95% CI: (2.3, 9.3)], duration on practical attachment [AOR = 2.6, 95% CI: (1.2, 5.9)], and knowledge about patient safety [AOR = 2.9, 95% CI: (1.9, 3.4)] were associated with better patient safety practices.

**Conclusion:**

In the current study the practice of patient safety among health science students was low and less than half of the students had good knowledge and favorable attitudes toward patient safety. Patient safety practices were influenced by the length of the clinical attachment, the study year, and the knowledge of patient safety. This calls for patient safety courses to be included in training curricula of undergraduate health sciences students.

## Introduction

Patient safety is minimizing the risk of unnecessary damage associated with healthcare to a minimum. A minimum is a sum of notions of given current information, available resources and the situation in which the provided healthcare is evaluated against the risk of no treatment or other treatment (1). Admitted patients run the chance of experiencing an adverse event, and those taking medications may face drug adverse reactions (2). According to current estimates, poor countries suffer more damage from unsafe medical care than developed countries do (3). Inclusion of lessons on patient safety for health professionals in higher educational programs is not at the required level (2).

According to a US research, medical mistakes cost $19.5 billion in lost revenue in only 2008 alone (4). According to a research from the Institute of Medicine, one of the most effective ways to increase patient safety is by integrating patient safety education into clinical training programs (5).

According to the WHO (4) report, the incidence of adverse events because of unsafe care is one of the 10 leading causes of mortality and disability globally. In low- and middle-income countries, about 134 million adverse events happen in hospitals because of unsafe care, which leads to 2.6 million deaths each year. Up to 4 in 10 patients are harmed in primary and outpatient health care globally, of which about 80% of the harm is avoidable (6). Medical errors and adverse events are a critical danger to patients globally (7). The evidence showed that ~98,000 persons die per year because of the medical errors that happen in hospitals. This is more than the death from motor vehicle accidents, workplace injuries, and breast cancer. Moreover, the financial burden of human tragedy and medical error easily increases to the peak ranks of urgent and widespread community issues (8).

Patient safety in Africa did not receive much attention until recently and was not even included in national policies, but now, thanks to the World Health Organization-African Partnership for Patient Safety, it has experienced a renaissance and is more and more recognized as a fundamental right within the framework of universal health coverage (9).

According to studies done at hospitals in the Oromia and Amhara regions about the level of KAP toward patient safety, there is a major lack of patient safety culture in Ethiopian public hospitals, with an overall level of patient safety culture of 46% (10, 11). Another study carried out in Dessie Town revealed patient safety culture level of < 50%. Working at primary hospitals was positively correlated with a good patient safety culture, whereas professionals between the ages of 25 and 34 and those employed in pediatrics and emergency rooms were negatively correlated (9). Patient safety culture was related to working hours, staffing levels, teamwork, open communications, reporting an incident, and sharing comments regarding errors (10, 11).

Despite a paucity of studies, patient safety in Ethiopia is thought to be a major problem. Nine point two (9.2) adverse medication events per 100 hospital admissions were found in a prior local investigation in the pediatric department; of these, almost 35% could have been avoided (12). Currently, as part of the Health Sector Transformation Plan (HSTP), Ministry of Health-Ethiopia is undertaking various initiatives to improve the quality of healthcare delivery. Despite the efforts by the Ministry of Health, there is a significant information gap regarding patient safety in Ethiopia. The provision of safe patient care is significantly impacted by the attitude, expertise, and practice of the health profession toward patient safety. In Ethiopia where primary prevention has long been a priority health policy, patient safety has been given due attention. Hence, the current study is aimed to assess knowledge, attitudes and practices regarding patient safety among undergraduate health students at Jimma University Institute of Health, southwest Ethiopia.

## Methods and materials

###  Study area and period

The study was conducted at Jimma University Institute of Health (JUIH). JUIH is located in Jimma Town, 352 km Southwest of Addis Ababa. The Town is home to various higher educational institutions as well as two government hospitals and three health facilities. The Jimma University Institute of Health has three faculties and nine disciplines, namely, medicine, dentistry, anesthesia, public health, pharmacy, medical laboratories, nursing, midwifery and environmental health. At the time of the study, there were 2,976 regular health science students at JUIH; of them, 1,555 have exposure to clinical attachment. The study was conducted from May 2021 to December 2021.

### Study design

An institution-based cross-sectional study design was employed.

### Source population

The source population for the study was all regular undergraduate health science students of JUIH.

### Study population

The study population was sampled regular undergraduate health science students who fulfilled the inclusion criteria.

#### Inclusion criteria

Medical interns, medical and dental students who were in fourth year and above, pharmacy students in fifth year, nursing, midwifery, public health officer, environmental health, anesthesia, and medical laboratory students who were above third year and all having at least 3 months of practical attachment at health facilities were included.

#### Exclusion criteria

Students who were sick at the time of data collection were excluded.

### Sample size determination and sampling technique

The total sample size was determined by using a single population proportion formula with a 95% confidence interval, marginal error (d) of 5% and *P* = 0.5, as there is no similar study to take the proportion assumed to be 50%. The final sample size was 678 after considering the correction formula, 10% non-response rate, and design effect of 2.

### Sampling technique

A stratified sampling technique was used, as the students were divided into different disciplines, and then the total sample size was proportionally allocated to each discipline. Then, the proportional sampling allocation method was used to select students in each of the class years within the discipline. Finally, a simple random sampling technique was employed to select study participants from each stratum using their list from the sampling frame. The total number of students with clinical attachment was 1,555.

### Data collection procedures

The WHO patient safety curriculum and patient safety guides were used as the basis for the development of the data collection instruments, which were then adapted to meet the study setting (2, 13). Some components of the questionnaire were directly adopted from research conducted among nurses working at Asella Referral and Teaching Hospital (14). The questionnaire had 18 questions on sociodemographic and personal-related characteristics, 11 knowledge questions, 9 attitude questions and 10 practice questions. The questionnaire was prepared in English. A semi-structured self-administered questionnaire was used to collect data from the selected students. A total of three BSc nurses, three GP data collectors and two resident physician supervisors were recruited for the study.

### Data quality control

To ensure the quality of the data, training was given to the data collectors, and the tools were pretested to determine their appropriateness and ethical soundness. The questionnaire was pretested on 5% of the sample size among students at Jimma University Medical Center (JUMC), which did not form part of the study participants. Close follow-up and supervision of the data collection process was carried out at every step of data collection. The principal investigator also cheeked the collected questionnaire for completeness and consistency every other day. The completed questionnaires were checked for completeness before entry.

### Data analysis

The collected data were checked, coded, and entered into EPI Data version 3.1 and then exported to SPSS version 25 for analysis. The outcome variables knowledge (poor knowledge and good knowledge), attitude (negative attitude and positive attitude), and practice (poor practice and good practice) were dichotomized. Then, these outcome variables were coded as knowledge (poor knowledge = 0 and good knowledge = 1), attitude (negative attitude = 0 and positive attitude = 1), and practice (poor practice = 0 and good practice = 1). Descriptive statistics were summarized by using tables, figures, and texts. Bivariable and multivariable logistic regression analyses were applied to identify variables associated with practice toward patient safety. The model fitness was checked by Hosmer–Lemeshow's goodness-of-fit test for practice toward patient safety, while the result was found to be (X^2^ = 14.458), (Df = 8), and (*P* = 0.071). The crude odds ratio and adjusted odds ratio with the corresponding 95% CI were calculated to show the strength of the association. Finally, variables in the multivariable logistic regression with *P* < 0.05 were considered statistically significant.

### Ethics approval and consent

The research was approved by the Institution of Health Research Ethics Review Committee of Jimma University before conducting the study. Support letter was obtained from Jimma University Institute of Health. For privacy and confidentiality reasons, personal identifiers were not used. The students were also informed that they had the right to withdraw from the study at any phase of the study. After obtaining full informed written consent from the participants, the study was performed using self-administered questionnaires.

### Operational definition

**Good knowledge**: If the respondents were able to answer 70% or more of the knowledge items.

**Good practice**: When the study participants were at least able to answer 70 % or more practice items correctly.

**Harm in healthcare**: Is any negative effect of patient care.

**Medical errors**: Are any preventable adverse effects of healthcare.

**Negative attitude**: If the respondents answered < 70% of attitude items.

**Patient management**: Interaction with the patient at any time from initiation to completion of treatment.

**Patient safety**: The reduction of risk of unnecessary harm associated with health care to an acceptable minimum.

**Poor knowledge**: If the respondents answered < 70% of the knowledge items.

**Poor practice**: When the participants were unable to answer 70 % of practice items correctly.

**Positive attitude**: If the respondents were able to give the correct answer for 70% or more of attitude items.

## Results

### Sociodemographic characteristics of students

The students' ages ranged from 20 to 32 years, with a mean of 23.9 (1.6) years. The bulk of the students, 465 (68.6%), were under the age of 25. More than half, 429 (63.3%), of them were male. Most, 649 (95.7%) of the students were single. Just over half 355 (52.4%) of them were medical students (Table 1).

**Table 1:** Sociodemographic characteristics of participants for assessment of knowledge, attitude and practice toward patient safety among undergraduate health science students of Jimma University, southwest Ethiopia, 2021.

**Variables**	**Category**	**Frequency**	**Percent**
Age	< 25	465	68.6
	≥25	213	31.4
Gender	Male	429	63.3
	Female	249	36.7
Religion	Muslim	226	33.3
	Orthodox	261	38.5
	Protestant	162	23.9
	Catholic	12	1.8
	Wakefeta	17	2.5
Marital status	Single	649	95.7
	Married	29	4.3
Discipline	Medicine	355	52.4
	Nursing	77	11.4
	Medical laboratory	58	8.6
	Dental medicine	30	4.4
	Health officer	45	6.6
	Midwifery	36	5.3
	Anesthesia	22	3.2
	Environmental health	23	3.4
	Pharmacy	32	4.7

### Attachment area and personal related characteristics

The majority, 263 (38.8%), of the students were in the fourth year. The clinical attachment area for most, 624 (92%), was at JUMC. Two hundred fifteen (31.7%) and 153 (22.6%) participants had 6–12 and 3–6 months of practical clinical attachment, respectively. Only 112 (16.5%) of the participants had formal training on patient safety. The majority of participants (457, 67.4%) had some information about patient safety. Only 142 (20.9%) students had their teachers (supervisors) encourage them to report medical errors (Table 2).

**Table 2:** Attachment area and related characteristics of participants for assessment of knowledge, attitude and practice toward patient safety among undergraduate health science students of Jimma University, southwest Ethiopia, 2021.

**Variables**	**Category**	**Frequency**	**Percent**
Year of Study	Third	111	16.4
	Fourth	263	38.8
	Fifth	174	25.6
	Sixth	130	19.2
For how long have you been in practical clinical attachment?	3–6 months	153	22.6
	6 months−1 year	215	31.6
	1 year−2 years	176	26
	>2 years	134	19.8
Have you ever heard of the term “Patient Safety”?	Yes	457	67.4
	No	221	32.6
Any previous formal education (training) about patient safety?	Yes	112	16.5
	No	566	83.5
Ever cared for or managed patients independently?	Yes	203	29.9
	No	475	70.1
Do teachers (supervisors) encourage reporting of medical errors?	Yes	142	20.9
	No	536	79.1
Current practical clinical attachment unit	Internal medicine	135	19.9
	Surgery	104	15.3
	Obstetrics and gynecology	132	19.5
	Pediatrics	96	14.2
	Laboratory unit	63	9.3
	ICU	15	2.2
	Dermatology/psychiatry/ ophthalmology (for clinical-2 medical students)	104	15.3
	Community attachment	24	3.5
	Pharmacy	5	0.8
Ever done harm on patients while practicing?	Yes	83	12.2
	No	595	87.8
Witnessed harm on patients by colleagues or other health workers	Yes	188	27.7
	No	490	72.3
Witnessed harm as a result of medical care to a close friend or family member?	Yes	102	15
	No	576	85

### Student's knowledge, attitude and practice toward patient safety

**Figure 1:**
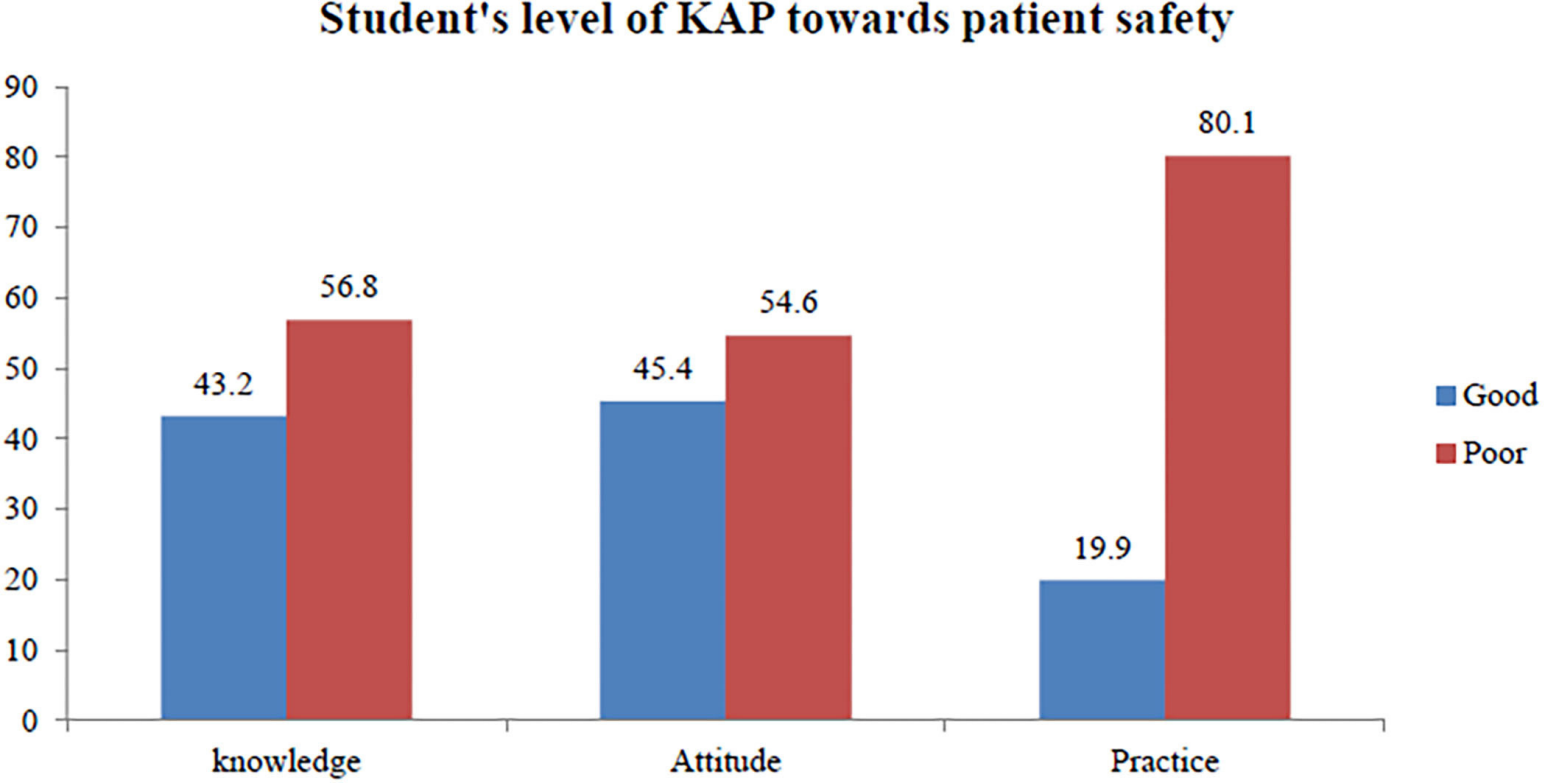
Level of knowledge, attitude and practice toward patient safety among health science students of Jimma University, Southwest Ethiopia, 2021 (*n* = 678).

The students' knowledge level about patient safety was found to be 43.2% [95% CI: (39.4, 47.2)]. Level of positive attitude toward patient safety was 54.6% [95% CI: (50.9, 58.6)], and the level of good practice toward patient safety was 19.9% [95% CI: (17, 23)] (Figure 1).

###  Factors associated with patient safety practice

Age, gender, residence, marital status, discipline, year of study, total duration of practical attachment, attachment unit, having information regarding patient safety, having formal or education (training) on patient safety, knowledge toward patient safety, and attitude toward patient safety were candidate variables for multivariable binary logistic regression in order to identify independent factors associated with patient safety practices. Accordingly, factors having a statistically significant association with patient safety practices were the year of study, total length of practical attachment, and knowledge of patient safety, with a *P* value 0.05. When compared to students in the third year of study, students in the fourth year of study were three times [AOR = 3, 95% CI: (1.2, 7.9)], those in the fifth year were twice [AOR = 1.9, 95% CI: (1.7, 6.2)], and those in the sixth year were three times [AOR = 3.75, 95% CI: (2.3, 9.3)] more likely to have good patient safety practices. The chance of having a good practice toward patient safety was 2.6 times [AOR = 2.6, 95% CI: (1.2, 5.9)] more among students who had a 6–12 month total duration of practical attachment than among students who were on their 3–6 month of practical attachment. Students who had good knowledge of patient safety were three times [AOR =2.9, 95% CI: (1.9, 3.4)] more likely to have good patient safety practices than students who had poor knowledge of patient safety (Table 3).

**Table 3:** A multivariate and bivariate logistic regression analysis of factors associated with practice toward patient safety among undergraduate health science students of Jimma University, southwest Ethiopia, 2021 (*n* = 678).

**Variables**	**Category**	**Practice**		**COR (95% CI)**	**AOR (95% CI)**	***P***
		**Good** ***N*** **(%)**	**Poor** ***N*** **(%)**			
Age	< 25	83 (17.8)	382 (82.2)	1	1	
	>25	161 (75.6)	52 (24.4)	1.6 (1.1,2.4)	1.1 (0.6, 1.8)	0.871
Residence	Urban	91 (22.3)	318 (77.7)	1.74 (1.1, 2.6)	1.3 (0.8, 2.1)	0.316
	Rural	44 (16.4)	225 (83.6)	1	1	
Discipline	Medicine	56 (15.8)	299 (84.2)	0.2 (0.1, 0.5)	0.26 (0.1, 1.2)	0.088
	Nursing	29 (37.7)	48 (62.3)	0.7 (0.3, 1.7)	2.9 (0.6, 13.3)	0.163
	Medical laboratory	3 (5.2)	55 (94.8)	0.07 (0.02, 0.3)	0.5 (0.07, 3.9)	0.547
	Dental medicine	8 (26.7)	22 (73.3)	0.5 (0.2, 1.4)	0.5 (0.1, 2.9)	0.414
	Health officer	7 (15.6)	38 (84.4)	0.24 (0.1, 0.7)	1.8 (0.3, 10.8)	0.530
	Midwifery	7 (19.4)	29 (80.6)	0.3 (0.1, 0.9)	1.4 (0.3, 7.2)	0.676
	Anesthesia	8 (36.4)	14 (63.6)	0.7 (0.24, 2.2)	3.9 (0.6, 25.6)	0.152
	Pharmacy	14 (43.7)	18 (56.3)	0.2 (0.05, 0.8)	1.6 (0.3, 9.3)	0.600
	Environmental health	3 (13)	20 (87)	1	1	
Year of study	3rd	11 (9.9)	100 (90.1)	1	1	
	4th	55 (20.7)	208 (79.3)	2.6 (1.3, 5.3)	3.0 (1.2, 7.9)	**0.024** ^*^
	5th	31 (17.8)	143 (82.2)	1.9 (0.9, 4.2)	1.92 (1.7, 6.2)	**0.003** ^*^
	6th	38 (29.2)	92 (70.8)	4.5 (2.1, 9.5)	3.75 (2.3, 9.3)	**0.004** ^*^
Total duration on attachment	3–6 month	27 (17.6)	126 (82.4)	1	1	
	6month−1 year	51 (23.7)	164 (76.3)	1.5 (0.9, 2.6)	2.6 (1.2, 5.9)	**0.018** ^*^
	1 year−2 years	14 (7.9)	162 (92.1)	0.4 (0.2, 0.8)	0.5 (0.2, 1.7)	0.306
	>2 years	43 (32.1)	91 (67.9)	2.6 (1.5, 4.5)	1.6 (0.4, 6.0)	0.496
Having formal training on patient safety	Yes	31 (27.7)	81 (72.3)	1.8 (1, 2.8)	0.8 (0.4, 1.5)	0.523
	No	104 (18.4)	462 (81.6)	1	1	
Have you ever cared for or managed patients independently	Yes	70 (34.5)	133 (65.5)	3.5 (2.3, 5.2)	0.6 (0.4, 1.1)	0.103
	No	65 (13.7)	410 (86.3)	1	1	
Teachers (Supervisors) encourage reporting	Yes	39 (27.5)	103 (72.5)	1.8 (1.2, 2.8)	0.9 (0.5, 1.6)	0.826
	No	96 (17.9)	440 (82.1)	1	1	
Knowledge toward patient safety	Good	86 (29.3)	207 (70.7)	2.8 (2.1, 4.6)	2.9 (1.9, 3.4)	**0.015** ^*^
	Poor	49 (12.7)	336 (87.3)	1	1	
Attitude toward patient safety	Good	44 (14.3)	264 (85.7)	2.2 (1.5, 3.3)	1.1 (0.6, 2.1)	0.648
	Poor	91 (24.6)	279 (75.4)	1	1	

^*^Bold value indicates statistically significant association.

## Discussion

Less than half of the students exhibited good knowledge and a favorable attitude toward patient safety, according to this survey. Furthermore, only few of the students had good patient safety practice. Students' practice of patient safety was highly influenced by their year of study, the length of their practical attachment, and their understanding of patient safety.

In the current study, students' good knowledge level toward patient safety was 43.2% [95% CI: (39.4, 47.2)]. This finding was lower than studies conducted in Ethiopia (15), Turkey (16), USA (17), Brazil (18), and Saudi Arabia (19) where knowledge toward patient safety were reported to be 48.4%, 50%, 58.4%, 89.8%, and 52.7%, respectively. A possible explanation for this variation could be the quality of teachers in monitoring and evaluation, medical equipment availability and availability of proper environment for teaching/training of patient safety. Moreover, the previous study only included nurses who worked as caretakers in hospitals; therefore, this variance may be caused by changes in sample size and the study population. According to this study, 54.6% of respondents had a favorable attitude toward patient safety [95% CI: (50.9, 58.6)]. The results of the current study is in line with those of a previous study that involved nurses in a tertiary hospital in Central Saudi Arabia, the University of Gondar Specialized Hospital, and Asella Referral and Teaching Hospital. In those studies, the levels of positive attitudes toward patient safety were 56.1%, 50.9%, and 57.9%, respectively (14, 15, 20). The study on KAP toward patient safety among nursing students in a private college in Malaysia found that 90.3% of students showed a positive attitude toward patient safety (21), and this result was higher than those obtained from studies on pharmacy students' attitudes in six developing nations in 2020, which found that 66.8% of them had a positive attitude toward patient safety (22). Additionally, the current study's findings were inferior to those of a study carried out at the University of Gondar, where 84.3% of participants reported feeling positively about patient safety (23). In contrast, this study finding was higher than that of a study conducted in Palestine Hospitals, which accounted for 42.8% (24).

This may be explained by the fact that the study in Malaysia only involved nursing students and was performed in a private college, which may have given it more weight because patient safety is a nurse's primary responsibility. Six different nations were represented in the other study, which may have highlighted the students' attitudes toward patient safety.

In the current study, the students' good practice level toward patient safety was only 19.9% [95% CI: 12, 23]. This result is lower than the study conducted among health professionals at referral hospitals in Asella and Gondar, Ethiopia, which reported 50% and 57.4% good practices, respectively (14, 15). The earlier investigations involved health professionals with extensive expertise as study participants, whereas the current study included students without any prior experience. This discrepancy may have resulted from this disparity. When compared to students in the third year of study, individuals who were in the fourth, fifth, and sixth years of study were three times, 1.9 times, and 3.75 times more likely to have good patient safety practices, respectively. This may be justified by the fact that attitudes toward patient safety will improve with years spent in contact with patients rise. This indicates that a focus on training and educational attainment is beneficial (25). Compared to students who had < 6 months of clinical attachment, students who had a total period of clinical attachment of 6 months or more were more likely to have good practices toward patient safety. When compared to students in their third year of study, those in their fourth or higher year of study showed better patient safety practices. The results of this study corroborate with prior studies' findings that greater nurse education levels are associated with better patient safety practices and outcome (26, 27). A possible explanation would be that students in their fourth and higher years spend more time in clinical attachments overall, which could boost their level of experience and practice.

Students who had better patient safety knowledge were more likely to practice good patient safety than students who had less patient safety knowledge. This is consistent with a systematic review on nurses' adherence to patient safety principles, which found that nurses in the USA exhibited a favorable correlation between knowledge and patient safety practice and healthcare professionals' knowledge as well as evidence-based medicine guidelines (18, 24). This might be because having good knowledge in the undergraduate program of patient safety would have a favorable impact on patient safety practice.

## Strength and limitations of the study

The strength of this study was that it included undergraduate health science students. However, being a single-center study and limited to regular health science students at Jimma University, the findings may not apply to all students studying health sciences in Ethiopia.

## Conclusion and recommendation

Only one-quarter of the students in the current study had good practice of patient safety, and less than half of the students had good knowledge and favorable attitudes toward patient safety. Patient safety practices were strongly influenced by the length of the clinical attachment, the study year, and the knowledge of patient safety. It is advised to include patient safety in the curricula of all medical and paramedical sciences and develop improved patient safety policies since students' lack of awareness about patient safety demonstrates the ineffectiveness of informal education.

## Data availability statement

The original contributions presented in the study are included in the article/supplementary material, further inquiries can be directed to the corresponding author.

## Ethics statement

The studies involving human participants were reviewed and approved by Institution of Health Research Ethics Review Committee of Jimma University. The patients/participants provided their written informed consent to participate in this study.

## Author contributions

The idea was conceived by TM, EG, EW, and MG, who also played significant roles in the proposal's development. TM, EG, EW, MG, and SK played a major role in the methodology and data interpretation. TM, EG, EW, MG, SK, and KA played a major role in the data analysis and discussion, contributed to writing, drafting, and editing the manuscript, and writing the discussion part. The analysis, writing, drafting, and editing of the book were done by all of the authors. Each author committed to be equally responsible for every part of the work after reading and giving final approval to the published version.

## References

[1] World Health Organization (WHO). Conceptual Framework for the International Classification for Patient Safety Version 1.1. Geneva: WHO (2009).

[2] World HealthOrganization (WHO). Patient Safety Curriculum Guide Multi-Professional Edition. Geneva: WHO (2009).

[3] Carpenter KB Duevel MA Lee PW Wu AW Bates DW Runciman WB. Methods and measures working group of the WHO world alliance for patient safety. Measures of patient safety in developing and emerging countries: a review of the literature. Qual Saf Health Care. (2010) 19:48–54. 10.1136/qshc.2008.031088 20172883

[4] WHO. Patient Safety. (2019). Available online at: https://www.who.int/news-room/fact-sheets/detail/patient-safety (accessed February 11, 2021).

[5] Van Den Bos J Rustagi K Gray T Halford M Ziemkiewicz E Shreve J. The $17.1 billion problem: the annual cost of measurable medical errors. Health Aff (Millwood). (2011) 30:596–603. 10.1377/hlthaff.2011.0084 21471478

[6] Schwappach D Conen D. Patient safety—who cares? Swiss Med Wkly. (2012) 142, w13634–w13634. 10.4414/smw.2012.13634 22802216

[7] Kohn LT, Corrigan, JM, Donaldson, MS, editors. To Err is Human: Building a Safer Health System. Washington (DC): National Academies Press (US) (2000). Available online at: http://www.ncbi.nlm.nih.gov/books/NBK225182/ (accessed February 11, 2021). 25077248

[8] World Health Organization (WHO). Partnership for Safer Health Service Delivery: Evaluation of WHO African Partnerships for Patient Safety 2009–2014. Geneva: WHO.

[9] Mekonnen AB McLachlan AJ Brien JA Mekonnen D Abay Z. Hospital survey on patient safety culture in Ethiopian public hospitals: a cross-sectional study. Saf Health. (2017) 3:11. 10.1186/s40886-017-0062-9

[10] Wami SD Demssie AF Wassie MM Ahmed AN. Patient safety culture and associated factors: a quantitative and qualitative study of healthcare workers' view in Jimma zone Hospitals, Southwest Ethiopia. BMC Health Serv Res. (2016) 16:495. 10.1186/s12913-016-1757-z 27644960 PMC5029028

[11] Mohammed F Taddele M Gualu T. Patient safety culture and associated factors among health care professionals at public hospitals in Dessie town, north east Ethiopia, 2019. PLoS ONE. (2021) 16:e0245966. 10.1371/journal.pone.0245966 33539368 PMC7861534

[12] Eshetie TC Hailemeskel B Mekonnen N Paulos G Mekonnen AB Girma T. Adverse drug events in hospitalized children at Ethiopian University Hospital: a prospective observational study. BMC Pediatr. (2015) 15:83. 10.1186/s12887-015-0401-0 26173560 PMC4502527

[13] World Health Organization (WHO): African Region. Guide for Developing National Patient Safety Policy and Strategic Plan. Geneva: WHO (2014).

[14] Wake AD Tuji TS Gonfa BK Waldekidan ET Beshaw ED Mohamed MA. Knowledge, attitude, practice and associated factors towards patient safety among nurses working at Asella Referral and Teaching Hospital, Ethiopia: a cross-sectional study. PLoS ONE. (2021) 16:e0254122. 10.1371/journal.pone.0254122 34197548 PMC8248719

[15] Biresaw H Asfaw N Zewdu F. Knowledge and attitude of nurses towards patient safety and its associated factors. Int J Africa Nurs Sci. (2020) 13:100229. 10.1016/j.ijans.2020.10022934197548

[16] Kerfoot BP Conlin PR Travison T McMahon GT. Patient safety knowledge and its determinants in medical trainees. J Gen Intern Med. (2007) 22:150–4. 10.1007/s11606-007-0247-8 17551796 PMC2305739

[17] Almaramhy H Al-Shobaili H El-Hadary K Dandash K. Knowledge and attitude towards patient safety among a group of undergraduate medical students in Saudi Arabia. Int J Health Sci (Qassim). (2011) 5:59–67. 22489231 PMC3312770

[18] Kiyancicek Z Dedeli O Yildiz E Senakin G. A survey: health professionals™ attitude towards: patient rights and patient safety. Asian J Pharm Nurs Med Sci. (2014) 2.

[19] Nabilou B Feizi A Seyedin H. Patient safety in medical education: students' perceptions, knowledge and attitudes. PLoS ONE. (2015) 10:e0135610. 10.1371/journal.pone.0135610 26322897 PMC4554725

[20] Alonazi NA Alonazi AA Saeed E Mohamed S. The perception of safety culture among nurses in a tertiary hospital in Central Saudi Arabia. Sudan J Paediatr. (2016) 16:51–8. 28096559 PMC5237835

[21] Vaismoradi M Tella SA Logan P Khakurel J Vizcaya-Moreno F. Nurses' adherence to patient safety principles: a systematic review. Int J Environ Res Public Health. (2020) 17:2028. 10.3390/ijerph17062028 32204403 PMC7142993

[22] Naser AY Alsairafi ZK Awaisu A Alwafi H Awwad O Dahmash EZ. Attitudes of pharmacy students towards patient safety: a cross-sectional study from six developing countries. BMJ Open. (2020) 10:e039459. 10.1136/bmjopen-2020-039459 33323431 PMC7745325

[23] Tegegn HG Abebe TB Ayalew MB Bhagavathula AS. Patient safety attitudes of pharmacy students in an Ethiopian university: a cross-sectional study. Drug Healthc Patient Saf. (2017) 9:19–24. 10.2147/DHPS.S128137 28507450 PMC5428765

[24] Elsous A Akbari Sari A Rashidian A Aljeesh Y Radwan M AbuZaydeh H. A cross-sectional study to assess the patient safety culture in the Palestinian hospitals: a baseline assessment for quality improvement. JRSM Open. (2016) 7:2054270416675235. 10.1177/2054270416675235 27928510 PMC5134298

[25] Kirwan M Matthews A Scott PA. The impact of the work environment of nurses on patient safety outcomes: a multi-level modelling approach. Int J Nurs Stud. (2013) 50:253–63. 10.1016/j.ijnurstu.2012.08.020 23116681

[26] Zulkifli NF Ahmad A Musa S Sinniah J Kunjukunju A. Perception, knowledge and attitude towards patient safety among nursing students in private college. Malaysian J Nurs. (2021) 13:68–76. 10.31674/mjn.2021.v13i01.010

[27] Hughes RG editor. Patient Safety and Quality: An Evidence-Based Handbook for Nurses. Prepared with support from the Robert Wood Johnson Foundation. AHRQ Publication No. 08-0043. Rockville, MD: Agency for Healthcare Research and Quality (2008). 21328752

